# Flooding and *Clostridium difficile* Infection: A Case-Crossover Analysis

**DOI:** 10.3390/ijerph120606948

**Published:** 2015-06-17

**Authors:** Cynthia J. Lin, Timothy J. Wade, Elizabeth D. Hilborn

**Affiliations:** 1 University of North Carolina, Gillings School of Global Public Health, Department of Epidemiology, Chapel Hill, NC 27599, USA; E-Mail: cjlin@email.unc.edu; 2 Oak Ridge Institute for Science and Education (ORISE) Research Participation Program at the U.S. Environmental Protection Agency, Chapel Hill, NC 27599, USA; 3 USA Environmental Protection Agency (EPA), Office of Research and Development, National Health and Environmental Effects Research Laboratory, Environmental Public Health Division, Research Triangle Park, NC 27709, USA; E-Mail: Hilborn.E@epa.gov

**Keywords:** epidemiology, *Clostridium difficile*, community-associated, flooding, case-crossover

## Abstract

*Clostridium difficile* is a bacterium that can spread by water. It often causes acute gastrointestinal illness in older adults who are hospitalized and/or receiving antibiotics; however, community-associated infections affecting otherwise healthy individuals have become more commonly reported. A case-crossover study was used to assess emergency room (ER) and outpatient visits for *C. difficile* infection following flood events in Massachusetts from 2003 through 2007. Exposure status was based on whether or not a flood occurred prior to the case/control date during the following risk periods: 0–6 days, 7–13 days, 14–20 days, and 21–27 days. Fixed-effects logistic regression was used to estimate the risk of diagnosis with *C. difficile* infection following a flood. There were 129 flood events and 1575 diagnoses of *C. difficile* infection. Among working age adults (19–64 years), ER and outpatient visits for *C. difficile* infection were elevated during the 7–13 days following a flood (Odds Ratio, OR = 1.69; 95% Confidence Interval, CI: 0.84, 3.37). This association was more substantial among males (OR = 3.21; 95% CI: 1.01–10.19). Associations during other risk periods were not observed (*p <* 0.05). Although we were unable to differentiate community-associated *versus* nosocomial infections, a potential increase in *C. difficile* infections should be considered as more flooding is projected due to climate change.

## 1. Introduction

### 1.1. Clostridium Difficile

*Clostridium difficile* is a spore-forming bacterium that can cause acute gastrointestinal symptoms (e.g., mild to moderate watery diarrhea, colitis without pseudomembrane formation, pseudomembranous colitis, fulminant colitis) [1]. It is considered the primary cause of nosocomial infectious diarrhea in the United States and other developed countries [2,3]. Since 2000, the rate of infection has continuously increased in the U.S., Canada, and Europe [1,4]. A 23% annual increase in U.S. hospitalizations for *C. difficile*-associated illness was detected from 2000 through 2005 [5]. The hospital discharge rate for *C. difficile* infection increased from less than 150,000 cases per year in 2000 to over 300,000 cases per year in 2006 [1,4]. As a result of mutations that increase the prevalence and virulence of the bacterium, infections have become more severe and difficult to treat [6,7]. In the U.S., there is no mandatory reporting of *C. difficile* infection; however, it is estimated that approximately 500,000 cases occur in U.S. hospitals and nursing homes per year [4,8]. In addition, 15,000 to 20,000 deaths have been attributed to *C. difficile* infection in the U.S. each year [1,4]. In 2009, data from the Healthcare Cost and Utilization Project revealed that 336,600 or nearly 1% of all U.S. hospital stays were related to *C. difficile* infection [9]. Of these, nearly a third had *C. difficile* infection as the principal diagnosis.

### 1.2. Community Transmission

*C. difficile* most often affects older adults who are hospitalized and receiving antimicrobial therapy [10,11]. However, the infection may also be community-associated, affecting otherwise healthy individuals who are not hospitalized or taking antibiotics [6,12,13,14,15]. In a random sample of Medicare beneficiaries, nearly half of those admitted with community-associated *C. difficile* had no recent antibiotic exposure [16]. Several studies have suggested an association between using proton pump inhibitors and increased risk for community-associated *C. difficile* infection [17,18]; however this association has been debated [19,20,21]. In a population-based study conducted in Minnesota, 41% of all *C. difficile* infections were community-associated [22]. These patients were generally younger, healthier, more likely to be female, and less likely to have a severe infection [22]. Therefore, reports of *C. difficile* infection among hospitalized patients may underestimate the burden of disease and overestimate severity [22,23,24]. Researchers investigating *C. difficile* infections in North Carolina concluded that 20% were community-associated and that the incidence rate was highest for middle-aged adults (45–64 years of age) and women [25]. Similarly, researchers in Sweden found that 22%–28% of *C. difficile* cases were community-associated [26,27]. In 2010, 32% of all cases identified across eight surveillance sites in the U.S. were community-associated [28].

### 1.3. Transmission in the Environment

Transmission of *C. difficile* occurs via the oral-fecal route [29,30]. If a symptomatic infected individual contaminates the surrounding environment, the infective spores can persist for several months [10]. The incubation period has been estimated at a median of 2 to 3 days, though it is not well-established [31]. The spores are resistant to heat and are resistant to some commonly used cleaning agents [32]. As a result, *C. difficile* can survive on surfaces for months and can be a continuous source of transmission in the absence of effective surface disinfection [33]. The persistence of the *C. difficile* may facilitate the spread of infection in the community [34].

People are potentially exposed to *C. difficile* exposure from multiple sources. Exposure to environmental reservoirs or sources of *C. difficile* such as food, air, water, and animals may play an important role in human infections [15,35,36]. *C. difficile* has been isolated from water samples collected from rivers, lakes, drainage channels, and the sea [29]. A diverse population of *C. difficile* has been observed in marine sediments*,* including isolates of ribotypes that have been associated with severe clinical infections [37]. In 2007, waterborne transmission of *C. difficile* spores was demonstrated after a drinking water distribution system in Finland was accidentally contaminated with sewage effluent from a municipal wastewater treatment plant [38].

### 1.4. Flooding and Gastrointestinal Infections

**Figure 1:**
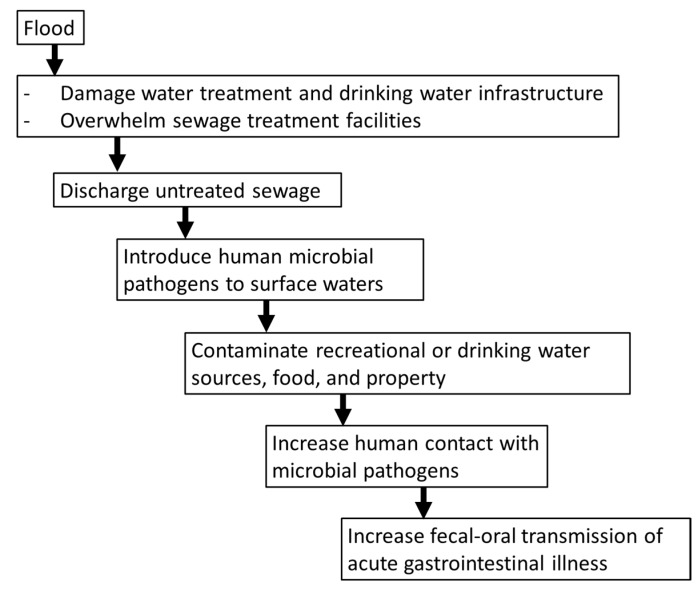
How flooding can facilitate the transmission of acute gastrointestinal infections.

Climate change has contributed to an increase in extreme weather events, including heavier rainfalls [39]. The northeastern part of the U.S. (e.g., Massachusetts to New Jersey) is particularly vulnerable to coastal flooding and the rise in sea level driven by climate change is projected to cause significant increases in storm surges [40]. As precipitation patterns change and heavier rainfall events occur, floods and droughts are likely to become more common and more intense [39,41,42]. Heavy downpours can overload drainage systems and water treatment facilities, increasing the risk for waterborne diseases [39,43,44]. Figure 1 illustrates the chain of events that may occur after a flood which could facilitate the transmission of acute gastrointestinal infections.

An increased incidence of gastrointestinal symptoms during a flood has been reported, as has an association between gastrointestinal symptoms and contact with floodwater [45]. In Massachusetts, an increased risk of visiting the emergency room for gastrointestinal symptoms has been reported during the first few days following a flood [46]. Of all emergency room visits for gastrointestinal symptoms during this period, an estimated 7% were attributable to flooding [46]. In an occupational cohort study of workers coming into contact with floodwater or sediment during a flood in Copenhagen, Denmark, diarrhea was the symptom of illness most frequently reported [47]. Also, during or shortly after a flood in Germany in 2002, individuals having direct contact with floodwater had an increased risk for diarrhea [48]. Aside from gastrointestinal illness, an increased risk of other disease outbreaks, such as hepatitis E and leptospirosis, have been observed after flood events [49]. To date, the impact of flooding on *C. difficile* infections has not yet been evaluated. The main objective of our study was to assess the impact of flooding on diagnoses of *C. difficile* infection at emergency room and outpatient settings in Massachusetts.

## 2. Methods

This study builds upon a recently published study that explored the association between flooding and emergency room (ER) visits for acute gastrointestinal illness, excluding *C. difficile* infection [46]. We use the same sources of data and we implement a similar case-crossover study design.

### 2.1. Heath Care Visits for Intestinal Infection due to C. Difficile

Massachusetts ER and hospital outpatient data were obtained for the years 2003 through 2007. Outpatient data included patients who received observation services and who were not admitted to the hospital. All data contained patient-level information, including town and zip code of residence, age, sex, diagnostic codes (International Classification of Disease, Version 9 Clinical Modification, ICD-9CM), and five associated diagnostic codes. Cases with primary or associated diagnosis of intestinal infection due to *C. difficile* were selected (ICD-9CM code 008.45).

These health data from the State of Massachusetts, Division of Health Care Finance and Policy, are yearly databases compiled from quarterly hospital reports and collected by the State for administrative purposes. They are made available to the public through an application process and do not contain information that could be used to identify an individual; the data are considered anonymous. These data were determined exempt from Institutional Review Board evaluation by the U.S. Environmental Protection Agency’s Human Subjects Research Protocol Officer as they were not considered human subjects data under the Common Rule (40 CFR 26).

### 2.2. Flood Events

Information on flood events occurring in the State of Massachusetts was obtained from the Storm Events Database maintained and compiled by the National Oceanic & Atmospheric Administration (NOAA), National Weather Service (http://www.ncdc.noaa.gov/stormevents/). Storms described with the event type “Flood”, which included “Coastal Floods”, “Flash Floods”, and related events were included in the analysis. Storms from December 2002 through January 2008 were included to allow for lagged and leading exposures for the health care visits and referent periods at the beginning and end of our five year study period. Information on floods included the start and end dates of the event and the location within a town (if available) or county.

### 2.3. Study Design

A case-crossover study design was used to assess the impact of flooding on ER and outpatient hospital visits for acute gastrointestinal illness due to *C. difficile* infection in Massachusetts. This type of study design is an efficient way to study transient exposures and acute health effects while controlling for fixed individual characteristics (e.g., sex, race) that do not vary with time [50]. Cases served as their own controls at a different referent time period before and/or after the disease diagnosis. Referent days were selected using a time-stratified bi-directional approach that matched on town (or county) and day of week [51]. Matching on day of week controlled for patterns in health care use by day of week. Time was stratified into 32 periods of 59 days. One or two referent periods were selected 4 weeks before and/or after the case, depending on when in the 59-day time period the case visit occurred. Restricting the referent selection to 4 weeks before and/or after the case controlled for any distinct seasonal patterns in both acute gastrointestinal illness and precipitation.

Exposure status was based on whether or not a flood occurred prior to the case/referent date during the following risk periods: 0–6 days, 7–13 days, 14–20 days, and 21–27 days. We allowed for extended risk periods (up to 27 days) to account for potential delays between the flood event, contamination of the environment, exposure, extended survival of spores (up to 70 days), and the median incubation period of the infection (2–3 days). Whenever possible, town was used to define exposure. When floods were described as county-wide, or no information was provided about the specific towns affected, all towns in the county were considered exposed. Health care visits were assigned to a town based on their ZIP code of residence and according to information from the U.S. Postal Service.

### 2.4. Statistical Analysis

Fixed-effects conditional logistic regression was used to estimate the risk of seeking health care for *C. difficile* infection following a flood. This type of regression model has been the standard for case-crossover studies [46,50,52,53] and it has also been shown to be unbiased when a time-stratified bi-directional approach is used for referent selection [51,54].

Results are reported as odds ratios (OR) and 95% confidence intervals (CI) and are interpreted as the relative increase in odds of diagnosis of *C. difficile* infection following a flood. The regression analysis was stratified by sex since community-associated *C. difficile* is reportedly more common among women [18,22,25,55] and we hypothesized that potential exposure to floodwaters may also differ by sex. The analysis was also stratified by age group (19–64, ≥65 years) since we hypothesized that direct flood water exposure may differ among working age adults and the elderly. Data management and statistical analyses were conducted using Stata SE Version 12 [56] and conditional logistic regression models were fit using the *xtlogit* command. Graphics were produced in Microsoft Office 2013 [57] and ArcGIS Desktop 10.2.1 [58].

## 3. Results

### 3.1. Health Care Visits for C. Difficile

Over a five year period from 2003 through 2007, there were 1575 diagnoses of *C. difficile* infections during ER and outpatient visits in the state of Massachusetts. Of those, 65% (*n* = 1023) were ER visits and 35% (*n =* 552) were outpatient. Table 1 describes these diagnoses by patient demographics and time. There were more females (69% ER; 62% outpatient) and the mean and median age were 59 and 63 years, respectively. Of all diagnoses, 49% occurred among the elderly (≥65 years), 46% among working age adults (ages 19–64 years), and 5% among children/youth (≤18 years). The majority (87%) of patients were non-Hispanic white. Just over half (59%) of all visits had a primary diagnosis for intestinal infection due to *C. difficile* and a quarter of all visits (27% ER; 22% outpatient) occurred on the weekend. Diagnoses varied by season with more occurring during the spring (27%) and summer (27%) than during the fall (23%) and winter (23%). The total number of diagnoses generally increased over time, ranging from 16% in 2004 to 25% in 2006 (Table 1).

**Table 1:** Diagnoses for *Clostridium difficile* at Massachusetts health care facilities during 2003–2007.

Diagnosis Characteristic	Emergency Room (ER)		Outpatient		ER + Outpatient	
	N = 1023		N = 552		N = 1575	
	N	%	*n*	%	*n*	%
Sex						
Female	708	69.2	341	61.8	1049	66.6
Male	315	30.8	211	38.2	526	33.4
Age group						
Children (≤5 years)	21	2.1	21	3.8	42	2.7
Youth (6–18 years)	26	2.5	17	3.1	43	2.7
Working Age Adults (19–64 years)	470	45.9	256	46.4	726	46.1
Elderly (≥65 years)	506	49.5	258	46.7	764	48.5
Race/ethnicity						
Non-Hispanic White	893	87.3	477	86.4	1370	87.0
Non-Hispanic Black	33	3.2	17	3.1	50	3.2
Hispanic	6	3.5	15	2.7	51	3.2
Other	7	1.7	10	1.8	7	1.7
Missing/Unknown	44	4.3	33	6.0	77	4.9
Primary diagnosis	597	58.4	338	61.2	935	59.4
Weekend visit	276	27.0	120	21.7	396	25.1
Season						
Fall	237	23.2	130	23.6	367	23.3
Winter	218	21.3	137	24.8	355	22.5
Spring	277	27.1	149	27.0	426	27.1
Summer	291	28.5	136	24.6	427	27.1
Year						
2003	174	17.0	86	15.6	260	16.5
2004	164	16.0	84	15.2	248	15.8
2005	190	18.6	126	22.8	316	20.1
2006	251	24.5	138	25.0	389	24.7
2007	244	23.9	118	21.4	362	23.0

### 3.2. Flood Events

**Figure 2:**
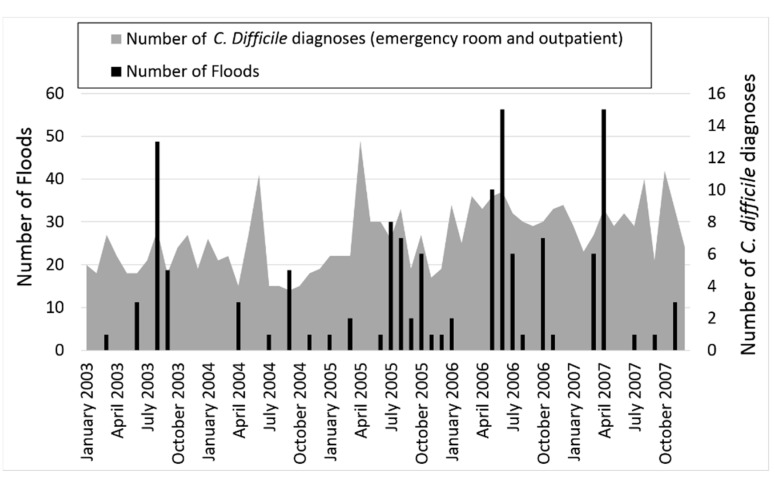
Flooding and *Clostridium difficile* diagnoses over time.

There were 129 flood events recorded in the NOAA database during 2003 through 2007 for the entire state of Massachusetts. These flood events occurred most often in the summer and least often in the winter (Table 2). They varied by year, ranging from 8% in 2004 to 33% in 2006. Figure 2 shows the monthly occurrence of flooding and *C. difficile* diagnoses during the entire study period.

**Table 2:** Flood events by season and year (N = 129).

	Number of Floods	%
Season		
Fall (September–November)	32	24.8
Winter (December–February)	4	3.1
Spring (March–May)	37	28.7
Summer (June–August)	56	43.4
Year		
2003	22	17.1
2004	10	7.8
2005	29	22.5
2006	42	32.6
2007	26	20.2

### 3.3. Association between Flooding and Diagnoses of Clostridium Difficile

We did not find an overall association between flooding and diagnosis of *C. difficile* infection diagnoses at health care facilities up to 27 days after flooding (Table 3). This remained the same after restricting to just the primary diagnoses. After stratifying by the predominant age groups (19–64 years; ≥65 years), we noticed a slightly elevated odds ratio among working age adults (19–64 years) during the 7–13 day period after flooding (OR = 1.69; 95% CI: 0.84–3.37).

**Table 3:** Association between flooding and diagnoses for *Clostridium difficile* at Massachusetts health care facilities.

Emergency Room or Outpatient Diagnosis ^1^	Number of Diagnoses	0–6 Days after Flooding	7–13 Days after Flooding	14–20 Days after Flooding	21–27 Days after Flooding
		OR (95% CI)	OR (95% CI)	OR (95% CI)	OR (95% CI)
Any *C. difficile*	1575	0.91 (0.54, 1.54)	1.32 (0.79, 2.20)	0.79 (0.50, 1.25)	1.23 (0.72, 2.09)
Primary diagnosis of *C. difficile*	935	1.02 (0.50, 2.08)	1.23 (0.65, 2.31)	0.65 (0.35, 1.22)	1.42 (0.71, 2.83)
Any *C. difficile*, Working Age Adults (19–64 years)	726	1.22 (0.60, 2.52)	1.69 (0.84, 3.37)	0.76 (0.39, 1.49)	1.12 (0.54, 2.33)
Any *C. difficile*, Elderly (≥65 years)	764	0.66 (0.29, 1.53)	0.86 (0.37, 1.99)	0.76 (0.40, 1.46)	1.30 (0.57, 2.96)
Any *C. difficile*, Males	526	1.03 (0.43, 2.49)	3.21 * (1.01, 10.19)	0.60 (0.28, 1.32)	1.28 (0.55, 2.97)
Any *C. difficile*, Females	1049	0.84 (0.44, 1.63)	1.00 (0.55, 1.81)	0.92 (0.51, 1.63)	1.20 (0.60, 2.38)

* *p <* 0.05. OR = Odds Ratio, 95% CI = 95% Confidence Interval; ^1^ Any diagnosis includes primary and associated diagnoses.

**Figure 3:**
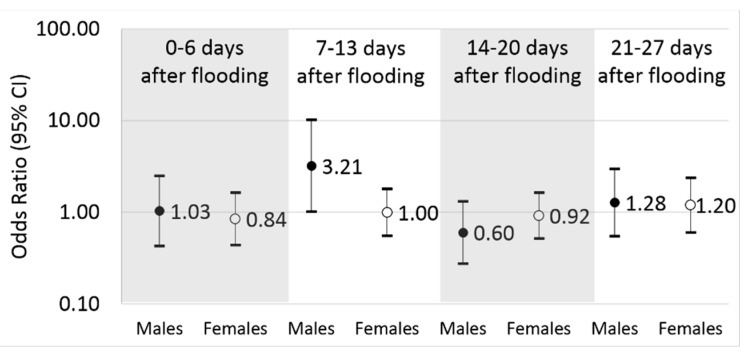
Association between flooding and health care visits for *Clostridium difficile*, by sex.

When we stratified the analyses by sex, we found that males were at an increased odds for diagnosis of *C. difficile* infection during the 7–13 day period after flooding (OR = 3.21; 95% CI: 1.01–10.19), compared to their referent periods (Table 3; Figure 3). Associations were not observed during other risk periods (0–6 days; 14–20 days; 21–27 days). Similar results for the 7–13 day risk period were found among males with only a primary diagnosis of infection (OR = 4.00; 95% CI: 1.09–14.70) and among working age adult males between the ages of 19 and 64 years (OR = 8.62; 95% CI: 1.05–71.00). Elderly males, however, did not have a statistically significant association (OR = 1.47; 95% CI: 0.33–6.66). There were no apparent associations among females for any risk period after flooding (Table 3; Figure 3).

## 4. Discussion

Using ER and hospital outpatient data from 2003 through 2007 for the entire state of Massachusetts, we found that males had an increased risk for diagnosis of *C. difficile* infection during the 7–13 day period after a flood event. This association remained among subsets of males, including working age adults between the ages of 19 and 64 and those with a primary diagnosis of *C. difficile* infection. We did not observe any other associations (e.g., during other risk periods, among females, overall) for diagnosis of *C. difficile* infections up to 27 days after flooding.

Finding a significant effect among males during the 7–13 day period after flooding seems plausible given the potential for long term survival of *C. difficile* spores in the environment and the median incubation period for *C. difficile* infection of 2–3 days after exposure. Since *C. difficile* spores are persistent [11,31], it is reasonable to think that infective spores could survive through a flood event and spread in the environment (e.g., food, water, air). After *C. difficile* enters the environment through flooding, it might take additional time to diagnosis. Only after ingestion can the spores germinate in the colon [59,60]. Considering the incubation period [31], it might be well over a week before an individual becomes symptomatic and seeks health care.

### 4.1. Differences by Sex

Although we only found an association with floods among males during the 7–13 day period after flooding, overall, health care visits for *C. difficile* infection were more common among females (67%). This is consistent with previous studies that have shown that community-associated *C. difficile* infections are more common in females [18,22,25,55]. One theory explaining this relates to more mothers than fathers regularly changing diapers of babies carrying *C. difficile* [15,61]. In terms of flooding, however, males may engage in more direct flood-associated labor [47,62], thereby receiving more contact with pathogens (e.g., *C. difficile*) and other potentially infective material in floodwaters or dried airborne debris containing infective spores. Heavy cleanup activities after the hurricanes in Louisiana in 2005 included power washing, removing drywall or carpeting, and moving furniture [63].

The difference in the observed effect between males and females may reflect differences in behavioral responses to floods by sex [64], meaning each gender overall may have differing degrees of flood water exposure or exposure to the recently flooded environment. Unfortunately, we did not have information about individual behaviors or activities to further specify degrees of flood exposure in our models. We also did not have occupational data to examine the risk of flood events among specific groups, such as sanitation/sewer workers or daycare workers, who may be more highly exposed to potentially infective material. After Hurricane Katrina in 2005, 80% of the relief workers injured were male [65]. Similarly, 84% of fatal work injuries caused by flooding in the U.S. between 1992 and 2006 occurred in males [66]. In an occupational cohort study in Copenhagen, Denmark, workers coming into contact with floodwater/sediment were predominantly males (87%) [47]. The proportions of floodwater-exposed workers experiencing subsequent illness, however, were similar (21% male; 24% female) [47]. Similarly, patients seen by a disaster medical assistance team following natural disasters in the U.S. did not differ greatly by sex, with 51% being female [67]. In contrast to our results, the risk for diarrhea was greater among women than among men during or shortly after a flood in Germany [48].

### 4.2. Age Group

Community-associated *C. difficile* infections, as opposed to nosocomial infections, often affects otherwise healthy individuals who are not hospitalized or taking antibiotics [6,12,13,14,15]. Among all ER and outpatient diagnoses of *C. difficile* infection in our study, 46% were among working age adults (19–64 years) and 49% were among the elderly (≥65 years). The significantly increased odds of diagnosed *C. difficile* infection observed among all males during the 7–13 day period after flooding (OR = 3.21; 95% CI: 1.01, 10.19) was further strengthened (although less precise) among working age adult males (*n* = 234; OR = 8.62; 95% CI: 1.05, 71.00). However, the statistically significant association (*p <* 0.05) of *C. difficile* infection with floods did not remain among elderly males (*n =* 255; OR = 1.47; 95% CI: 0.33–6.66). This could be due to differences in flood exposure between working age adult and elderly males. It seems plausible that working age adults would have more direct flood involvement than the elderly during cleanup activities and everyday activities (e.g., going to work, running errands). After Hurricane Katrina in 2005, over 70% of injuries among residents and relief workers occurred among those between the ages of 15 to 64 years and only 11% occurred among the elderly (≥65 years) [65].

### 4.3. Study Strengths and Limitations

Since there is currently no mandatory reporting of *C. difficile* infection in the U.S. [68], there are challenges to estimating incidence of infection within populations. Our analysis was able to take advantage of daily and town-level data for *C. difficile* diagnoses and flooding. By using ICD-9 diagnostic codes, we were able to identify *C. difficile* infections in a way that could be replicated at other hospitals and healthcare facilities [68]. Dubberke *et al.* (2006) report that diagnostic codes correlate well with *C. difficile*-toxin assay results (sensitivity 78%, specificity 99.7%) [68]. As with any administrative database, a limitation is that only a subset of *C. difficile* infections are ascertained since among some infected individuals, symptoms may not be severe enough to require immediate medical attention. Without capturing the less severe infections, we may have underestimated any true association between flooding and *C. difficile* infection.

Although *C. difficile* often affects older adults who are hospitalized and receiving antimicrobial therapy, some of the cases in our study (especially the 46.1% of working age adults) could represent community-associated infections since they were diagnosed at the time of their ER or outpatient visit *versus* during a hospitalization. In our study, however, we were unable to differentiate between community-associated and nosocomial infections. We cannot evaluate the role of established risk factors for infection since we do not have individual information on previous hospitalizations, residence at nursing home or long term care facility, or use of antibiotic and over-the-counter drugs. Also, depending on the severity of the flood, it is even possible that affected residents were provided antibiotics to care for physical wounds sustained from their clean-up efforts [67]. Nevertheless, given the persistence of *C. difficile* [34] and its ability to be transmitted in water [38], it is plausible that people were exposed to *C. difficile* directly or indirectly via contaminated floodwaters. For example, one route of potential exposure is for healthcare workers and visitors to contaminate healthcare facilities (e.g., hospitals, nursing homes, long term care facility) with pathogens carried in from the external environment [69,70].

Our case-crossover study design controlled for individual characteristics that do not vary during the 59 day stratified time periods used for referent selection. These characteristics include sex, race, age, socioeconomic status, and any underlying health conditions. Although diagnoses varied by season, with more occurring during the warmer months, the differences were small (<5%). As a result, selecting referent periods 4 weeks before and/or after the *C. difficile* diagnosis should adequately control of any potential confounding by seasonality. By matching referent days on the day of week, we accounted for potential differences in health care seeking behaviors by day. We used a time-stratified approach to referent selection in order to avoid overlap bias and time trend bias in the exposure [51,54]. A limitation to our referent selection was that, without personally identifiable information, we could not confirm that a case did not have a different ER or outpatient visit during his/her referent period. We expect these cases to have been rare and that any resulting misclassification would be random in relation to flooding exposure and would therefore not result in a systematic bias of the results. We made assumptions regarding lags and incubation periods for this study, and as a result, we may have missed flood-associated *C. difficile* diagnoses that occurred longer than 27 days after a flood.

A limitation to our flood definition is that all flood events were treated equally. We did not have detailed enough information from the Storm Events Database to consider the severity or type of flood. Also, due to the study design, we only accounted for the onset (start date) of flooding and not the duration. The majority (*n =* 102; 79%) of floods, however, lasted only 1 day. Another limitation to our flood definition was that, for the flood events that were deemed county-wide or else missing information about affected towns, we had to assign all towns in the county as exposed. This could have resulted in non-differential exposure misclassification, which would have the effect of biasing our estimates towards the null thereby reducing the magnitude of the association between flooding and diagnosis of *C. difficile* infection. However, for the majority of flood events (*n =* 91; 71%), we had town-level information.

Although we observed an elevated risk of *C. difficile* infection among males after flooding, the small number of cases in the stratified groups (e.g., 315 males with a primary diagnosis; 234 working age adult males) decreased the precision of the effect estimates. Relatively few cases and a small effect size could have made any other association (e.g., during other lag periods, among other subpopulations), if present, difficult to identify.

## 5. Conclusions

This study used Massachusetts ER and hospital outpatient data to evaluate diagnosis of *C. difficile* infection following flood events. Our results suggest that males may be at an increased risk for *C. difficile* infection during the 7–13 day period following flood events. This association should be considered when flooding causes environmental contamination. We did not observe any other associations (e.g., during other risk periods, among females, overall) for *C. difficile* infections up to 27 days after a flood. With more floods events projected to occur in the future as a result of climate change, a small increased risk in *C. difficile* infections may become more important as larger numbers of people are exposed to flood waters and recently flooded environments.

## References

[1] Kachrimanidou M., Malisiovas N. (2011). *Clostridium difficile* infection: A comprehensive review. Crit. Rev. Microbiol..

[2] Kyne L., Farrell R.J., Kelly C.P. (2001). Clostridium difficile. Gastroenterol. Clin. N. Am..

[3] Guerrant R.L., Hughes J.M., Lima N.L., Crane J. (1990). Diarrhea in developed and developing-countries—Magnitude, special settings, and etiologies. Rev. Infect. Dis..

[4] Rupnik M., Wilcox M.H., Gerding D.N. (2009). *Clostridium difficile* infection: New developments in epidemiology and pathogenesis. Nat. Rev. Microbiol..

[5] Zilberberg M.D., Shorr A.F., Kollef M.H. (2008). Increase in adult *Clostridium difficile*-related hospitalizations and case-fatality rate, united states, 2000–2005. Emerg. Infect. Dis..

[6] Kelly C.P., LaMont J.T. (2008). *Clostridium difficile*—More difficult than ever. N. Engl. J. Med..

[7] Todd B. (2006). *Clostridium difficile*: Familiar pathogen, changing epidemiology. Am. J. Nurs..

[8] O’Donoghue C., Kyne L. (2011). Update on *Clostridium difficile* infection. Curr. Opin. Gastroenterol..

[9] Lucado J., Gould C., Elixhauser A. Clostridium Difficile Infections (CDI) in Hospital Stays, 2009: Statistical Brief #124. http://www.hcup-us.ahrq.gov/reports/statbriefs/sb124.pdf.

[10] Centers for Disease Control and Prevention Healthcare-Associated Infections (HAIS): Clostridium Difficile Infection. http://www.cdc.gov/hai/organisms/cdiff/cdiff_infect.html.

[11] Barbut F., Petit J.C. (2001). Epidemiology of *Clostridium difficile-*associated infections. Clin. Microbiol. Infect..

[12] Centers for Disease Control and Prevention (2005). Severe *Clostridium difficile*-associated disease in populations previously at low risk—Four states, 2005. MMWR.

[13] Pituch H. (2009). *Clostridium difficile* is no longer just a nosocomial infection or an infection of adults. Int. J. Antimicrob. Agents.

[14] Kuijper E.J., van Dissel J.T. (2008). Spectrum of *Clostridium difficile* infections outside health care facilities. CMAJ Can. Med. Assoc. J..

[15] Gupta A., Khanna S. (2014). Community-acquired *Clostridium difficile* infection: An increasing public health threat. Infect. Drug Resist..

[16] Collins C.E., Ayturk M.D., Flahive J.M., Emhoff T.A., Anderson F.A., Santry H.P. (2014). Epidemiology and outcomes of community-acquired *Clostridium difficile* infections in Medicare beneficiaries. J. Am. Coll. Surg..

[17] Deshpande A., Pant C., Pasupuleti V., Rolston D.D., Jain A., Deshpande N., Thota P., Sferra T.J., Hernandez A.V. (2012). Association between proton pump inhibitor therapy and *Clostridium difficile* infection in a meta-analysis. Clin. Gastroenterol. Hepatol..

[18] Chitnis A.S., Holzbauer S.M., Belflower R.M., Winston L.G., Bamberg W.M., Lyons C., Farley M.M., Dumyati G.K., Wilson L.E., Beldavs Z.G. (2013). Epidemiology of community-associated *Clostridium difficile* infection, 2009 through 2011. JAMA Intern. Med..

[19] Naggie S., Miller B.A., Zuzak K.B., Pence B.W., Mayo A.J., Nicholson B.P., Kutty P.K., McDonald L.C., Woods C.W. (2011). A case-control study of community-associated *Clostridium difficile* infection: No role for proton pump inhibitors. Am. J. Med..

[20] Tleyjeh I.M., Bin Abdulhak A.A., Riaz M., Alasmari F.A., Garbati M.A., AlGhamdi M., Khan A.R., Al Tannir M., Erwin P.J., Ibrahim T. (2012). Association between proton pump inhibitor therapy and *Clostridium difficile* infection: A contemporary systematic review and meta-analysis. PLoS ONE.

[21] Freedberg D.E., Abrams J.A. (2013). *Clostridium difficile* infection in the community: Are proton pump inhibitors to blame?. World J. Gastroenterol. WJG.

[22] Khanna S., Pardi D.S., Aronson S.L., Kammer P.P., Orenstein R., St Sauver J.L., Harmsen W.S., Zinsmeister A.R. (2012). The epidemiology of community-acquired *Clostridium difficile* infection: A population-based study. Am. J. Gastroenterol..

[23] Khanna S., Baddour L.M., Huskins W.C., Kammer P.P., Faubion W.A., Zinsmeister A.R., Harmsen W.S., Pardi D.S. (2013). The epidemiology of *Clostridium difficile* infection in children: A population-based study. Clin. Infect. Dis..

[24] Wilcox M.H., Mooney L., Bendall R., Settle C.D., Fawley W.N. (2008). A case-control study of community-associated *Clostridium difficile* infection. J. Antimicrob. Chemother..

[25] Kutty P.K., Woods C.W., Sena A.C., Benoit S.R., Naggie S., Frederick J., Evans S., Engel J., McDonald L.C. (2010). Risk factors for and estimated incidence of community-associated *Clostridium difficile* infection, North Carolina, USA. Emerg. Infect. Dis..

[26] Karlstrom O., Fryklund B., Tullus K., Burman L.G. (1998). A prospective nationwide study of Clostridium difficile-associated diarrhea in Sweden. The Swedish *C. difficile* study group. Clin. Infect. Dis..

[27] Noren T., Akerlund T., Back E., Sjoberg L., Persson I., Alriksson I., Burman L.G. (2004). Molecular epidemiology of hospital-associated and community-acquired *Clostridium difficile* infection in a Swedish county. J. Clin. Microbiol..

[28] Lessa F.C. (2013). Community-associated *Clostridium difficile* infection: How real is it?. Anaerobe.

[29] Al Saif N., Brazier J.S. (1996). The distribution of *Clostridium difficile* in the environment of South Wales. J. Med. Microbiol..

[30] Centers for Disease Control and Prevention Resources for Patients: Clostridium Difficile Infection. http://www.cdc.gov/hai/organisms/cdiff/Cdiff-patient.html.

[31] Cohen S.H., Gerding D.N., Johnson S., Kelly C.P., Loo V.G., McDonald L.C., Pepin J., Wilcox M.H., Society for Healthcare Epidemiology of American, Infectious Diseases Society of American (2010). Clinical practice guidelines for *Clostridium difficile* infection in adults: 2010 Update by the Society for Healthcare Epidemiology of America (SHEA) and the Infectious Diseases Society of America (IDSA). Infect. Control Hosp. Epidemiol..

[32] Wilcox M.H., Fawley W.N. (2000). Hospital disinfectants and spore formation by *Clostridium difficile*. Lancet.

[33] Kramer A., Schwebke I., Kampf G. (2006). How long do nosocomial pathogens persist on inanimate surfaces? A systematic review. BMC Infect. Dis..

[34] Nazarko L. (2014). *Clostridium difficile* in the community: Time to clean up?. Br. J. Community Nurs..

[35] Best E.L., Sandoe J.A., Wilcox M.H. (2012). Potential for aerosolization of *Clostridium difficile* after flushing toilets: The role of toilet lids in reducing environmental contamination risk. J. Hosp. Infect..

[36] Hoover D.G., Rodriguez-Palacios A. (2013). Transmission of *Clostridium difficile* in foods. Infect. Dis. Clin. N. Am..

[37] Hargreaves K.R., Colvin H.V., Patel K.V., Clokie J.J., Clokie M.R. (2013). Genetically diverse *Clostridium difficile* strains harboring abundant prophages in an estuarine environment. Appl. Environ. Microbiol..

[38] Kotila S.M., Pitkanen T., Brazier J., Eerola E., Jalava J., Kuusi M., Kononen E., Laine J., Miettinen I.T., Vuento R. (2013). *Clostridium difficil* contamination of public tap water distribution system during a waterborne outbreak in Finland. Scand. J. Public Health.

[39] U.S. Global Change Research Program Global Climate Change Impacts in the United States. http://purl.access.gpo.gov/GPO/LPS116712.

[40] Kirshen P., Watson C., Douglas E., Gontz A., Lee J., Tian Y. (2008). Coastal flooding in the Northeastern United States due to climate change. Mitig. Adapt. Strateg. Glob. Chang..

[41] Knox J.C. (1993). Large increases in flood magnitude in response to modest changes in climate. Nature.

[42] Milly P.C.D., Wetherald R.T., Dunne K.A., Delworth T.L. (2002). Increasing risk of great floods in a changing climate. Nature.

[43] McMichael A.J., Woodruff R.E., Hales S. (2006). Climate change and human health: Present and future risks. Lancet.

[44] Lane K., Charles-Guzman K., Wheeler K., Abid Z., Graber N., Matte T. (2013). Health effects of coastal storms and flooding in urban areas: A review and vulnerability assessment. J. Environ. Public Health.

[45] Wade T.J., Sandhu S.K., Levy D., Lee S., LeChevallier M.W., Katz L., Colford J.M. (2004). Did a severe flood in the Midwest cause an increase in the incidence of gastrointestinal symptoms?. Am. J. Epidemiol..

[46] Wade T.J., Lin C.J., Jagai J.S., Hilborn E.D. (2014). Flooding and emergency room visits for gastrointestinal illness in Massachusetts: A case-crossover study. PLoS ONE.

[47] Wojcik O.P., Holt J., Kjerulf A., Muller L., Ethelberg S., Molbak K. (2013). Personal protective equipment, hygiene behaviours and occupational risk of illness after July 2011 flood in Copenhagen, Denmark. Epidemiol. Infect..

[48] Schnitzler J., Benzler J., Altmann D., Mucke I., Krause G. (2007). Survey on the population’s needs and the public health response during floods in Germany 2002. J. Public Health Manag. Pract. JPHMP.

[49] Alderman K., Turner L.R., Tong S. (2012). Floods and human health: A systematic review. Environ. Int..

[50] Maclure M. (1991). The case-crossover design: A method for studying transient effects on the risk of acute events. Am. J. Epidemiol..

[51] Janes H., Sheppard L., Lumley T. (2005). Case-crossover analyses of air pollution exposure data: Referent selection strategies and their implications for bias. Epidemiology.

[52] Maclure M., Mittleman M.A. (2000). Should we use a case-crossover design?. Ann. Rev. Public Health.

[53] Mittleman M.A., Maclure M., Robins J.M. (1995). Control sampling strategies for case-crossover studies: An assessment of relative efficiency. Am. J. Epidemiol..

[54] Janes H., Sheppard L., Lumley T. (2005). Overlap bias in the case-crossover design, with application to air pollution exposures. Stat. Med..

[55] Kuntz J.L., Chrischilles E.A., Pendergast J.F., Herwaldt L.A., Polgreen P.M. (2011). Incidence of and risk factors for community-associated *Clostridium difficile* infection: A nested case-control study. BMC Infect. Dis..

[56] StataCorp LP (2011). Stata Statistical Software, Version SE12.

[57] Microsoft (2013). Microsoft Office 2013.

[58] ESRI (2014). Arcgis for Desktop, Version 10.2.1.

[59] Martinez F.J., Leffler D.A., Kelly C.P. (2012). *Clostridium difficile* outbreaks: Prevention and treatment strategies. Risk Manag. Healthc. Policy.

[60] Paredes-Sabja D., Shen A., Sorg J.A. (2014). *Clostridium difficile* spore biology: Sporulation, germination, and spore structural proteins. Trends Microbiol..

[61] Leffler D.A., Lamont J.T. (2012). Editorial: Not so nosocomial anymore: The growing threat of community-acquired *Clostridium difficile*. Am. J. Gastroenterol..

[62] Fothergill A. (1999). Women’s roles in a disaster. Appl. Behav. Sci. Rev..

[63] Riggs M.A., Rao C.Y., Brown C.M., Van Sickle D., Cummings K.J., Dunn K.H., Deddens J.A., Ferdinands J., Callahan D., Moolenaar R.L. (2008). Resident cleanup activities, characteristics of flood-damaged homes and airborne microbial concentrations in New Orleans, Louisiana, October 2005. Environ. Res..

[64] Mustafa D. (2003). Reinforcing vulnerability? Disaster relief, recovery, and response to the 2001 flood in Rawalpindi, Pakistan. Glob. Environ. Chang. Part B Environ. Hazards.

[65] Sullivent E.E., West C.A., Noe R.S., Thomas K.E., Wallace L.J., Leeb R.T. (2006). Nonfatal injuries following Hurricane Katrina—New Orleans, Louisiana, 2005. J. Saf. Res..

[66] Fayard G.M. (2009). Fatal work injuries involving natural disasters, 1992–2006. Disaster Med. Public Health Prep..

[67] Nufer K.E., Wilson-Ramirez G., Shah M.B., Hughes C.E., Crandall C.S. (2006). Analysis of patients treated during four disaster medical assistance team deployments. J. Emerg. Med..

[68] Dubberke E.R., Reske K.A., McDonald L.C., Fraser V.J. (2006). Icd-9 Codes and surveillance for *Clostridium difficile*-associated disease. Emerg. Infect. Dis..

[69] Hsieh Y.H., Liu J., Tzeng Y.H., Wu J. (2014). Impact of visitors and hospital staff on nosocomial transmission and spread to community. J. Theor. Biol..

[70] Alam M.J., Anu A., Walk S.T., Garey K.W. (2014). Investigation of potentially pathogenic *Clostridium difficile* contamination in household environs. Anaerobe.

